# Supplementary material for: Coumarin-Containing Light-Responsive Carboxymethyl Chitosan Micelles as Nanocarriers for Controlled Release of Pesticide
Source: Polymers (Basel). 2020 Oct 1;12(10):2268. doi: 10.3390/polym12102268 (PMC7601645; doi:10.3390/polym12102268)
Supplement: Supplementary file 1 [file polymers-12-02268-s001.pdf]

Supplementary Materials

Coumarin-Containing Light-Responsive Carboxymethyl Chitosan Micelles as Nanocarriers for Controlled Release of Pesticide

Song Feng, Junqin Wang, Lihua Zhang *, Qin Chen, Wang Yue, Ke Ni and Haibo Xie *

Department of Polymer Materials and Engineering, College of Materials and Metallurgy, Guizhou University, Guiyang 550025, China

* Correspondence: lhzhang5@gzu.edu.cn (L.Z.); hbxie@gzu.edu.cn (H.X.)

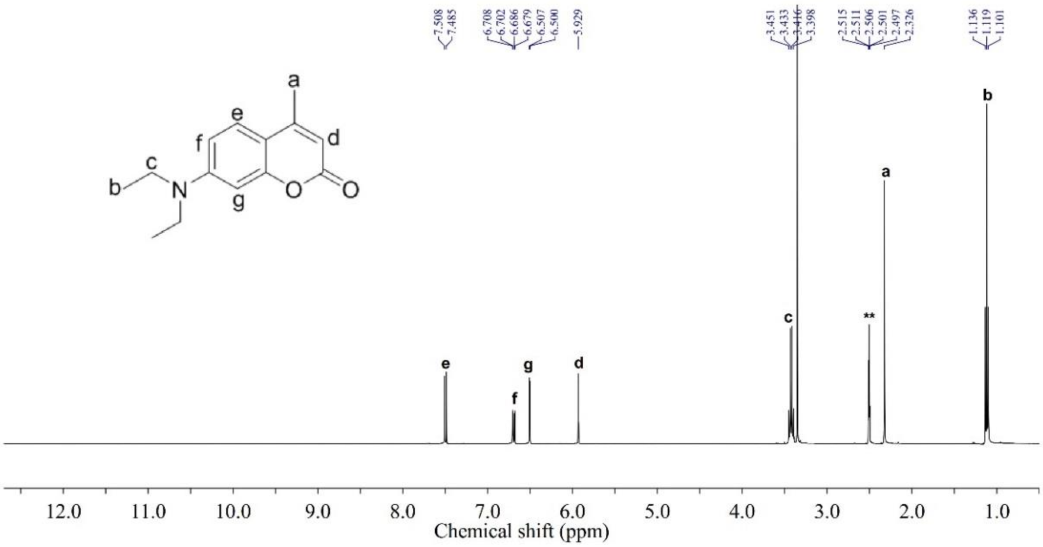

Figure S1. ^{1}H NMR spectrum of 7-diethylamino-4-methylcoumarin in DMSO-d_6 (**).

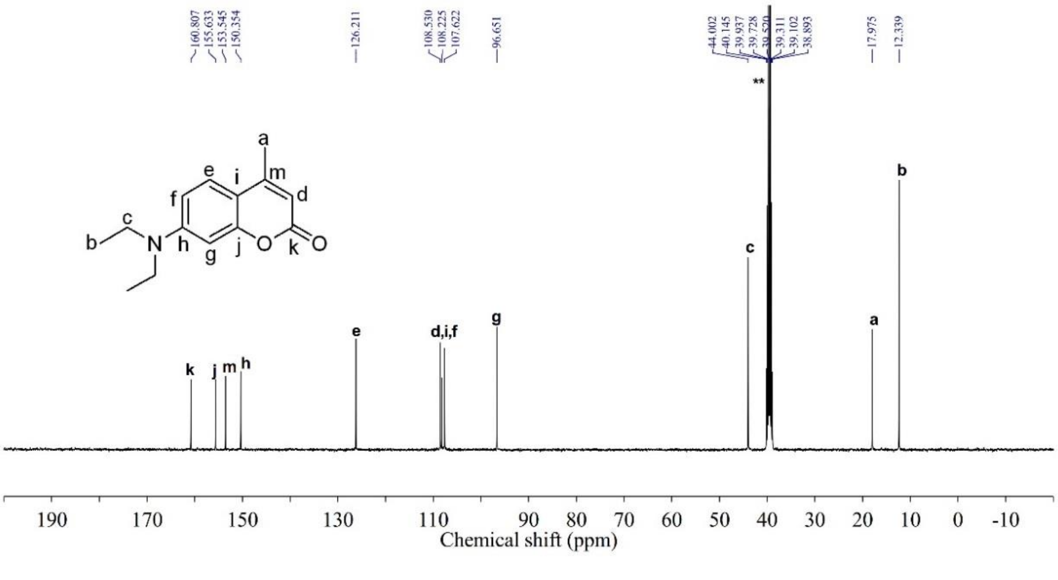

Figure S2. ^{13}C NMR spectrum of 7-diethylamino-4-methylcoumarin in DMSO-d_6 (**).

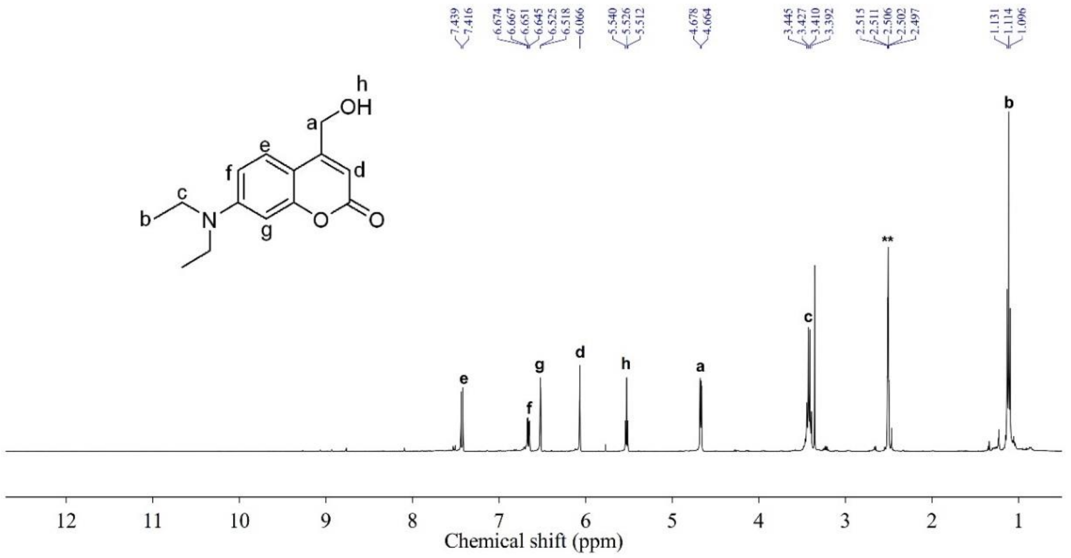

Figure S3. ^{1}H NMR spectrum of 7-diethylamino-4-hydroxymethylcoumarin (**1**) in DMSO-d_6 (**).

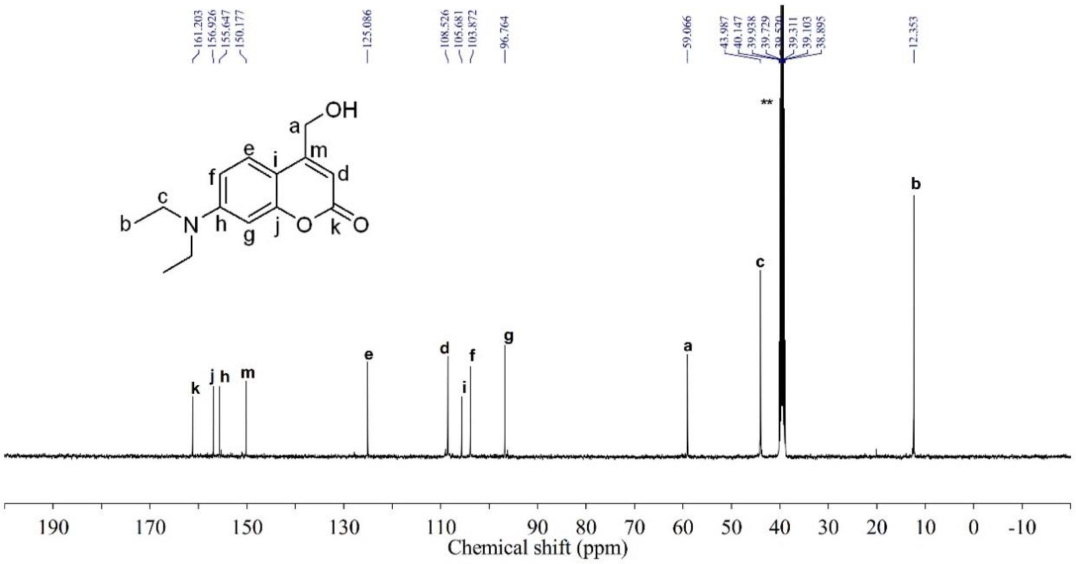

Figure S4. ^{13}C NMR spectrum of 7-diethylamino-4-hydroxymethylcoumarin (**1**) in DMSO-d_6 (**).

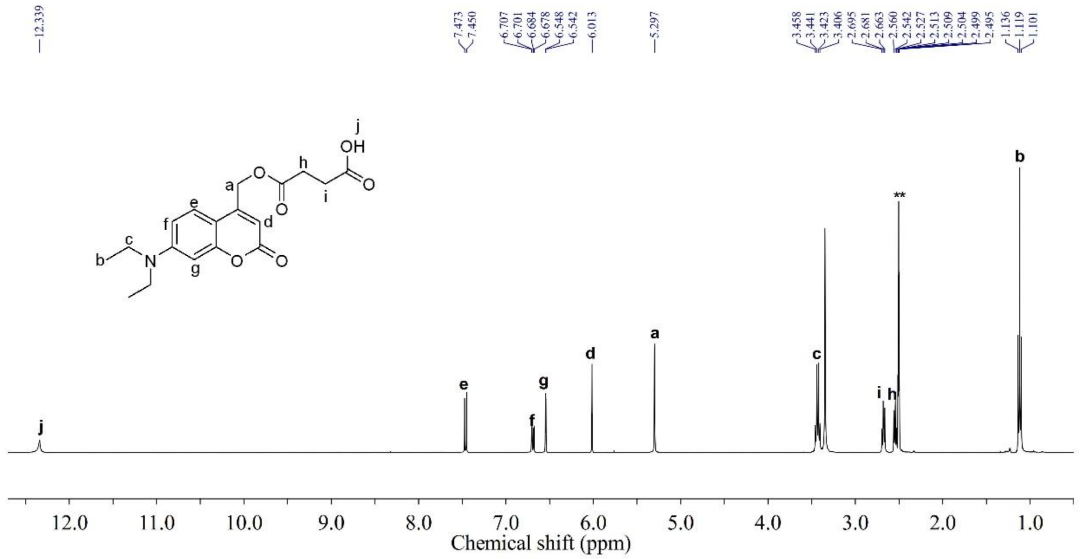

Figure S5. ^{1}H NMR spectrum of DEACMS in DMSO-d_6 (**).

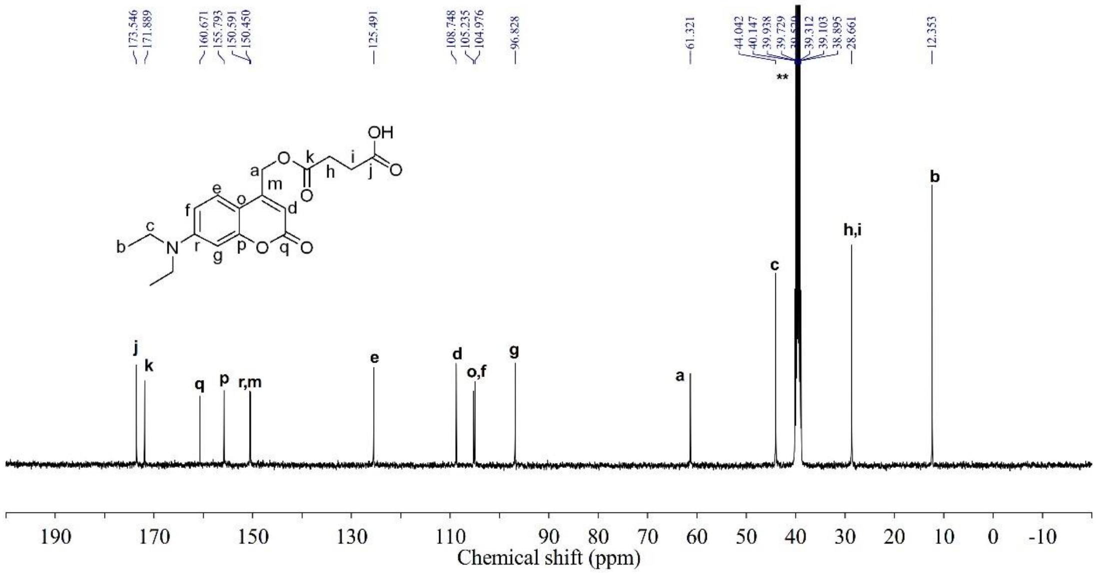

Figure S6. ^{13}C NMR spectrum of DEACMS in DMSO-d_6 (**).

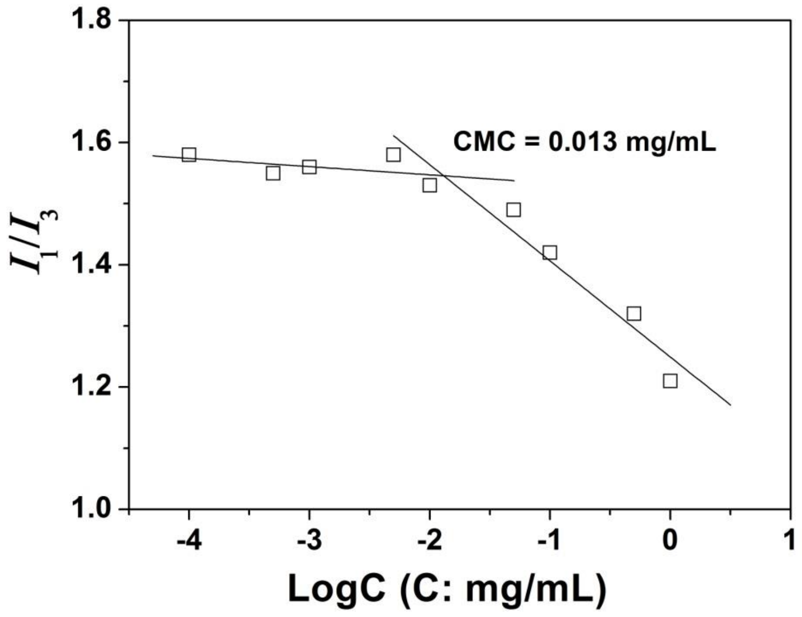

Figure S7. The intensity ratio (I_1/I_3) of pyrene emission spectra versus the logarithm concentration of CMCS-DEACMS-1.

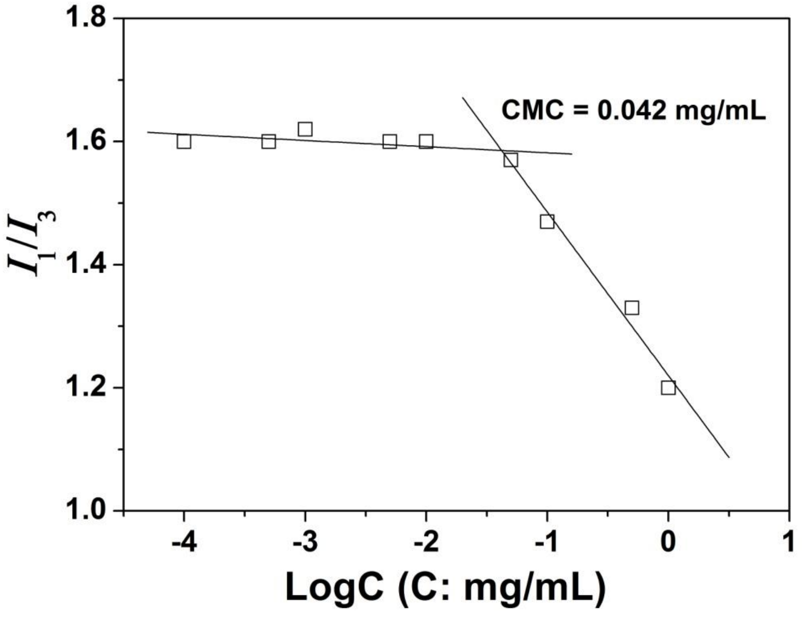

Figure S8. The intensity ratio (I_1/I_3) of pyrene emission spectra versus the logarithm concentration of CMCS-DEACMS-3.

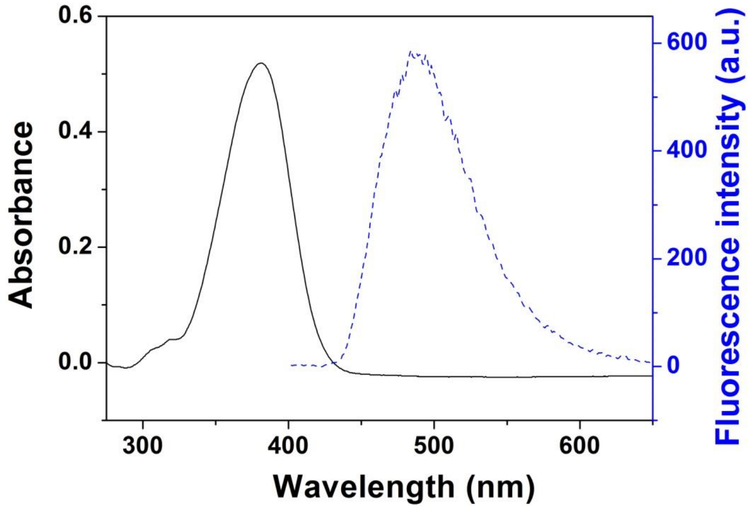

Figure S9. UV-vis (solid line) and emission (dashed line) spectra of DEACMS in DMSO.

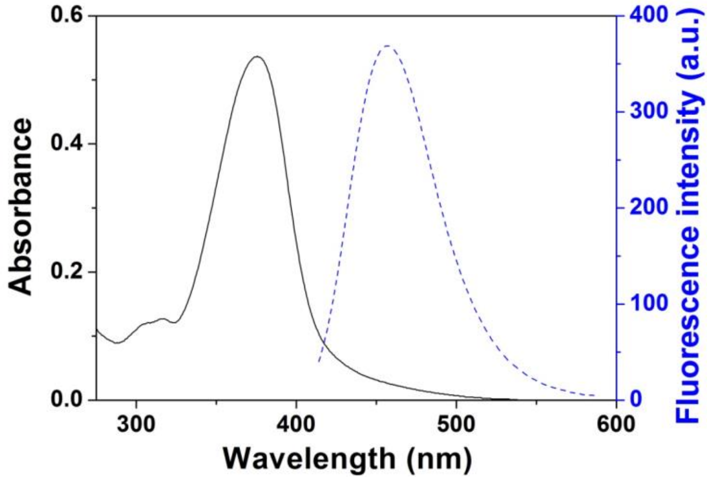

Figure S10. UV-vis (solid line) and emission (dashed line) spectra of compound **1** in DMSO.

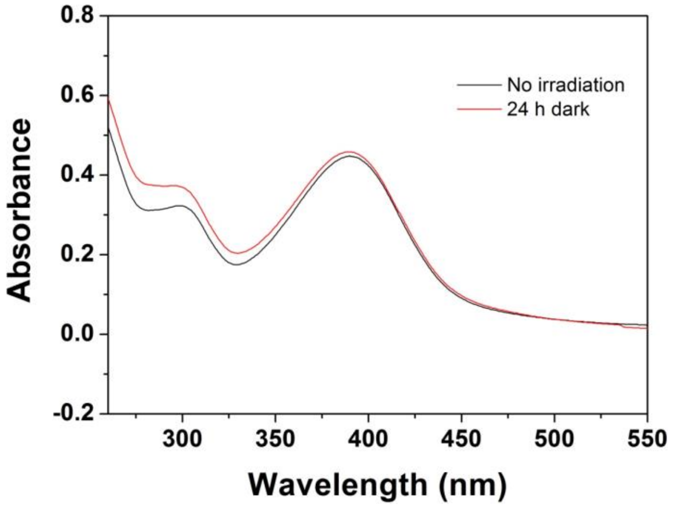

Figure S11. UV-vis absorption spectra of CMCS-DEACMS-2 micelles in aqueous solution kept in the dark.

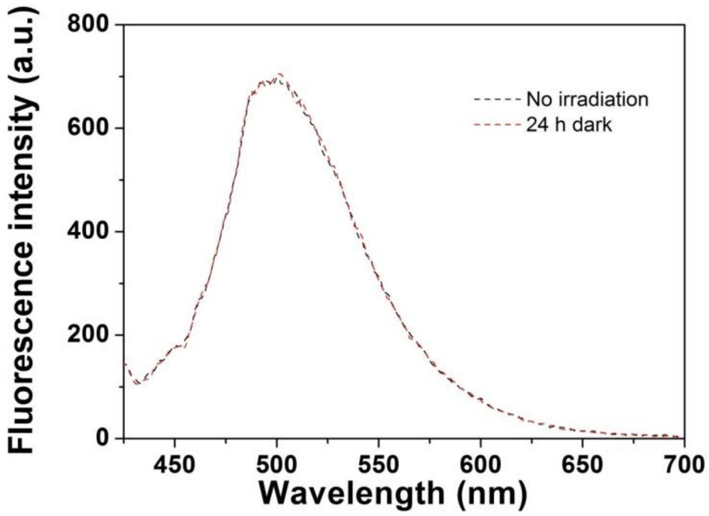

Figure S12. Emission spectra of CMCS-DEACMS-2 micelles in aqueous solution kept in the dark.

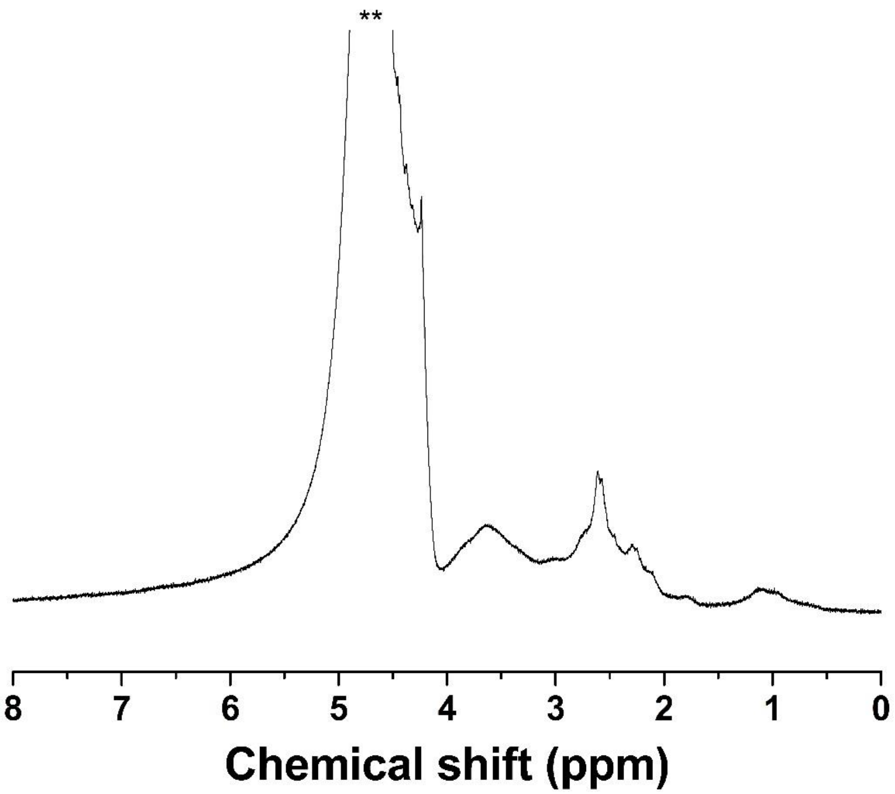

Figure S13. ^{1}H NMR assessment of the photolytic product in D_2O after irradiation under simulated sunlight.
